# Dual Regulation of Voltage-Sensitive Ion Channels by PIP_2_

**DOI:** 10.3389/fphar.2012.00170

**Published:** 2012-09-25

**Authors:** Aldo A. Rodríguez-Menchaca, Scott K. Adney, Lei Zhou, Diomedes E. Logothetis

**Affiliations:** ^1^Department of Physiology and Biophysics, School of Medicine, Virginia Commonwealth UniversityRichmond, VA, USA

**Keywords:** PIP2, voltage sensor, voltage-gated channels, gating, S4–S5 linker

## Abstract

Over the past 16 years, there has been an impressive number of ion channels shown to be sensitive to the major phosphoinositide in the plasma membrane, phosphatidylinositol 4,5-bisphosphate (PIP_2_). Among them are voltage-gated channels, which are crucial for both neuronal and cardiac excitability. Voltage-gated calcium (Cav) channels were shown to be regulated bidirectionally by PIP_2_. On one hand, PIP_2_ stabilized their activity by reducing current rundown but on the other hand it produced a voltage-dependent inhibition by shifting the activation curve to more positive voltages. For voltage-gated potassium (Kv) channels PIP_2_ was first shown to prevent N-type inactivation regardless of whether the fast inactivation gate was part of the pore-forming α subunit or of an accessory β subunit. Careful examination of the effects of PIP_2_ on the activation mechanism of Kv1.2 has shown a similar bidirectional regulation as in the Cav channels. The two effects could be distinguished kinetically, in terms of their sensitivities to PIP_2_ and by distinct molecular determinants. The rightward shift of the Kv1.2 voltage dependence implicated basic residues in the S4–S5 linker and was consistent with stabilization of the inactive state of the voltage sensor. A third type of a voltage-gated ion channel modulated by PIP_2_ is the hyperpolarization-activated cyclic nucleotide-gated (HCN) channel. PIP_2_ has been shown to enhance the opening of HCN channels by shifting their voltage-dependent activation toward depolarized potentials. The sea urchin HCN channel, SpIH, showed again a PIP_2_-mediated bidirectional effect but in reverse order than the depolarization-activated Cav and Kv channels: a voltage-dependent potentiation, like the mammalian HCN channels, but also an inhibition of the cGMP-induced current activation. Just like the Kv1.2 channels, distinct molecular determinants underlied the PIP_2_ dual effects on SpIH, with the proximal C-terminus implicated in the inhibitory effect. The dual regulation of these very different ion channels, all of which are voltage-dependent, points to conserved mechanisms of regulation of these channels by PIP_2_.

## Introduction

Voltage-gated ion channels regulate the flow of different ions across the membrane in response to changes in membrane potential. These channels are composed of four subunits (or four linked domains) symmetrically arranged around a central ion-conducting pore. Voltage-gated ion channels open in response to depolarization or hyperpolarization. The change in membrane potential induces a conformational change in the voltage sensor domain located in the periphery of the ion channel; this conformational change is coupled to the ion-conducting pore, and leads to channel opening (Long et al., 2005; Bezanilla, 2008; Borjesson and Elinder, 2008).

Voltage-gated ion channels can be modulated by numerous factors including free fatty acids (Xiao et al., 2005; Borjesson et al., 2008; Xu et al., 2008a), toxins (Catterall et al., 2007; Swartz, 2007), metal ions (Elinder and Arhem, 2003), glycosylation (Fozzard and Kyle, 2002; Watanabe et al., 2003, 2007), palmitoylation (Gubitosi-Klug et al., 2005; Jindal et al., 2008), phosphorylation (Davis et al., 2001; Mohapatra and Trimmer, 2006; Mohapatra et al., 2007; Li et al., 2008), and phospholipids (Ramu et al., 2006; Schmidt et al., 2006; Xu et al., 2008b). Modulation by phospholipids includes phosphatidylinositol 4,5-bisphosphate (PIP_2_), the lipid component of the inner membrane leaflet that modulates the activity of most ion channels and transporters tested (Suh and Hille, 2005, 2008; Logothetis et al., 2010).

PIP_2_ plays an important role as an intermediate molecule in multiple receptor signaling pathways. PIP_2_ hydrolysis by PLC produces 1,4,5-trisphosphate (IP_3_) and diacylglycerol (DAG; Berridge, 1984). IP_3_ mobilizes Ca^2+^ from the endoplasmic reticulum, while DAG activates PKC. However, PIP_2_ itself acts as a signaling molecule through direct interactions with target proteins. Our review focuses on the effects of PIP_2_ on voltage-dependent ion channels, particularly those showing bidirectional regulation.

## Voltage-Gated Calcium Channels

Voltage-gated calcium (Cav) channels mediate calcium influx in response to membrane depolarization and regulate intracellular processes such as contraction, secretion, neurotransmission, and gene expression in many different cell types. There are five types of Cav channels: the high voltage-activated L- (Cav1), P/Q- (Cav2.1), N- (Cav2.2), and R- (Cav2.3), and the low voltage-activated T- (Cav3; Catterall, 2011). Cav channels are complexes of α1, α2, β, γ, and δ subunits. It is in the α1 subunit that the conduction pore and voltage sensor apparatus are located. The α1 subunit is composed of four homologous domains (I–IV), with six transmembrane segments (S1–S6) in each. Similar to Kv and voltage-gated sodium (Nav) channels, the S1–S4 segments serve as the “voltage sensor” and the S5–S6 segments form the “pore domain” (Catterall et al., 2005).

Voltage-gated Ca^2+^ channels of the Cav2 subfamily (N- and P/Q-type) are regulated by G protein coupled receptors via two distinct pathways in sympathetic neurons (Ikeda and Dunlap, 1999). The first is the faster pathway, voltage-dependent, and membrane delimited, induced by direct interaction of the G protein βγ subunit with the channel protein (Herlitze et al., 1996; Ikeda, 1996; Dolphin, 2003). The second is the slower pathway that is voltage-independent. It uses signals that stimulate the G_q/11_ type of G proteins to activate phospholipase C (PLC), which hydrolyzes PIP_2_ into inositol triphosphate (IP_3_) and diacylglycerol (DAG; Bernheim et al., 1991; Brown et al., 1997). This slower pathway was later attributed to depletion of PIP_2_ by the activation of PLC (Wu et al., 2002; Gamper et al., 2004). The faster pathway has also been related to PIP_2_. Rousset et al. (2004) reported that decreasing PIP_2_ levels suppressed the constitutive inhibition of Cav2.1 channels by endogenous Gβγ subunits. These authors proposed that stabilization of the Gβγ sensitive state of Cav2.1 channels may require direct interaction with PIP_2_. However, additional studies are needed to firmly establish the importance of PIP_2_ in this pathway.

The first study on Cav channel modulation by PIP_2_ reported that this phosphoinositide induces two opposing modulatory effects on Cav2.1 channels (Wu et al., 2002). Rundown of Cav2.1 channels in inside-out patches of *Xenopus* oocytes was greatly slowed and even reversed by the application of exogenous PIP_2_ or Mg-ATP to the patch. Conversely, the application of PIP_2_-antibody accelerated rundown, suggesting that PIP_2_ stabilizes the activity of Cav2.1 channels and its depletion induces rundown. PIP_2_ also exerted a voltage-dependent inhibitory effect by shifting the voltage dependence of activation toward depolarized potentials. This effect was antagonized by activation of protein kinase A (PKA; Wu et al., 2002). Modulation of Cav2.1 channels by PIP_2_ has also been reported to occur in neostriatal projection neurons. Stimulation of muscarinic M1 receptors inhibited Cav2.1 channels in these neurons, an effect that could be abolished by inhibition of PLC. Consistent with these results, intracellular application of PIP_2_ inhibited all muscarinic modulation of Cav2.1 channels (Perez-Burgos et al., 2010).

In subsequent studies, Cav2.2 modulation by PIP_2_ was also reported (Gamper et al., 2004). In inside-out *Xenopus* oocytes patches expressing Cav2.2 channels, PIP_2_ significantly slowed or reversed the rundown of these channels. In native Cav2.2 channels from sympathetic neurons, the current inhibition by muscarinic M1 receptor activation was diminished by intracellular application of diC8-PIP_2_, and the current recovery was abolished when PIP_2_ synthesis was blocked. Interestingly, activation of bradykinin receptors, which also activate PLC and induce PIP_2_ hydrolysis, failed to inhibit Cav2.2 currents in the same neurons in which muscarinic M1 receptor activation was effective, an effect attributed to a probable concurrent Ca^2+^-mediated stimulation of PIP_2_ synthesis (Gamper et al., 2004). In a different study with the same type of neurons, bradykinin-induced voltage-independent inhibition of Ca^2+^ channels was reported and this effect could be abolished by inhibiting PLC, but it was not altered by inhibiting its downstream effectors (Lechner et al., 2005). The discrepancies with the previous study (i.e., Gamper et al., 2004) were attributed by the authors to differences in experimental conditions, such as the use of different cell culture media, differences in the buffering of intracellular Ca^2+^ concentrations, differences in intracellular Mg^2+^, and differences in the voltage protocols used.

Another hypothesis for the slow G_q/11_ mediated inhibition of L-, N-, and P/Q calcium channels involves arachidonic acid (AA). G_q/11_ coupled receptor stimulation can acutely activate PLA_2_ with the subsequent production of AA, which is proposed as the main signal mediating Cav channels inhibition (Roberts-Crowley et al., 2009). Thus, the same receptors that induce PIP_2_ depletion can cause concurrent release of AA and modulate Cav channels according to the AA hypothesis.

A recent elegant study used two strategies to deplete PIP_2_ without the production of the PLC downstream products (Suh et al., 2010). PIP_2_ was depleted by rapamycin-induced translocation of an inositol lipid 5-phosphatase and a voltage-sensitive 5-phosphatase (VSP). These systems convert PI(4,5)P_2_ to PI(4)P in the plasma membrane of intact cells, without activation of G protein-coupled receptors. Both systems suppressed Cav1.2, Cav1.3, Cav2.1, and Cav2.2 channels. Irreversible depletion of endogenous PIP_2_ by rapamycin-induced translocation of INP54p 5-phosphatase to the plasma membrane irreversibly inhibited whole-cell Cav currents. On the other hand, reversible depletion of PIP_2_ by the activation of the zebrafish voltage-sensitive phosphatase *Danio rerio* (Dr-VSP) reversibly inhibited Cav channels in whole-cell recordings, suggesting that PIP_2_ is a cofactor required for channel activity. These results with intact cells (whole-cell experiments) did not completely recapitulate the effects reported in inside-out patches with Cav2.1 and Cav2.2 channels (Wu et al., 2002; Gamper et al., 2004). While in excised patches currents ran down almost completely, in intact cells PIP_2_ depletion inhibited Cav2.1 currents by 29% and Cav2.2 by 55%. In addition, the inhibitory actions of PIP_2_ were not observed. These differences as suggested by the authors could potentially reflect preservation in the whole-cell recordings of phosphorylation in some channels or cytoplasmic factors that make channels less PIP_2_ sensitive or preserving PIP_2_ synthesis that prevents full PIP_2_ depletion (Suh et al., 2010).

Recently, it was demonstrated that the β subunits of voltage-gated Ca^2+^ channels also influence regulation by PIP_2_ (Suh et al., 2012). Cav channels co-expressed with the β3 subunit could be partially inhibited by activating a voltage-sensitive lipid phosphatase to deplete PIP_2_ (Suh et al., 2010). However, when these channels were co-expressed with the β2a subunit, the inhibition was smaller (Suh et al., 2012). The palmitoylation of two cysteine residues in the N terminus of the β2a subunit was responsible for this decrease in inhibition of Cav channel activity. When the palmitoylation sites were mutated, the β2a subunit behaved more like a β3 subunit. Furthermore, addition of a lipidation motif to β3 subunits reduced the inhibitory effects of Cav channels, similar to the palmitoylated β2a subunit (Suh et al., 2012). Thus, Cav channel regulation by PIP_2_ is dependent on both the α and β subunits. Previously, a similar pattern of β subunit effects on arachidonic acid modulation of Cav channels was reported (Heneghan et al., 2009; Mitra-Ganguli et al., 2009).

## Voltage-Gated Potassium Channels

Voltage-gated potassium (Kv) channels are involved in diverse physiological processes, including action potential repolarization, secretion of hormones and neurotransmitters, contraction of skeletal muscle, and others. Kv channels are homotetrameric, with each subunit containing the S1–S4 voltage sensor domain and the S5–S6 central pore domain (Yellen, 2002). As with Cav channels, the movement of the voltage sensor domain in response to membrane depolarization initiates conformational changes that lead to the pore opening. After channel opening, Kv channels undergo a time-dependent loss of conductivity by a mechanism termed inactivation. Two distinct mechanisms of inactivation have been described, N-type (or “ball and chain”) inactivation, in which the N-terminal domain of certain α or β subunits of Kv channels plugs the open channel pore from the cytoplasmic side (Hoshi et al., 1991), and C-type inactivation, which appears to result from constriction of the selectivity filter (Yellen, 1998).

Only a few studies have demonstrated PIP_2_ involvement in the regulation of voltage-dependent K^+^ channels. PIP_2_ shows remarkable effects on the N-type inactivation of certain voltage-gated K^+^ channels, specifically, Kv1.4 and Kv3.4 (in which the “ball domain” is located in the N terminus of the α pore-forming subunit) and Kv1.1 co-expressed with the “ball domain”-containing Kvβ1.1 accessory subunit (Oliver et al., 2004). Application of exogenous PIP_2_ to the intracellular side of the membrane expressing these channels removed the rapid N-type inactivation, regardless of whether the “ball domain” resided at the N terminus of the channel α or β subunit. It was proposed that PIP_2_ insertion into the plasma membrane immobilized the positively charged “ball domain” through its negatively charged head-group and thereby prevented it from accessing the open pore. In this study a cluster of positive residues formed by Arg^13^ and Lys^14^ in the “ball domain” of Kv3.4 were proposed as the place where the electrostatic interaction between the PIP_2_ and “ball domain” occurs (Oliver et al., 2004). Later, these findings were repeated for Kv1.5 channels co-expressed with the Kvβ1.3 accessory subunit. PIP_2_ eliminated the Kvβ1.3 induced inactivation of Kv1.5 channels by immobilization of its “ball domain” as reported previously. An Arginine residue (Agr^5^) in the N terminus of Kvβ1.3 subunit was identified as a critical residue for PIP_2_ binding to the channel (Decher et al., 2008). Thus, changes in intracellular PIP_2_ levels might be important for the inactivation of Kv channels and this would profoundly alter electrical signaling.

Shab channels, a prototypical member of the Kv2 channels subfamily (Wei et al., 1990) are also modulated by PIP_2_. It was shown that standard light stimulation of *Drosophila* photoreceptors increased Shab currents (Krause et al., 2008). After light stimulation, the voltage dependence of activation of Shab channels was shifted to hyperpolarized potentials about 10 mV, with a small decrease on the current amplitude at depolarized potentials. The mechanism proposed for this modulation of Shab channels involves the activation of PLCβ4 and the resulting hydrolysis of PIP_2_. Interestingly, a point mutation (R435Q) in the N terminus of the Shab channel abolished the PIP_2_ hydrolysis effect. The same results were obtained expressing recombinant Shab channels in *Drosophila* S2 cells and recording currents in inside-out patches. Within a few minutes of patch excision the threshold for activation was left-shifted about 10 mV, an effect that was almost completely reversed by application of diC8-PIP_2_ to the patch. These results led Krause et al. (2008) to suggest that PIP_2_ may interact directly with the Shab channel and modulate its activity.

Kv1.3 is another voltage-gated K^+^ channel reported to be modulated by PIP_2_. The Kv1.3 channel is important in the activation and function of effector memory T cells (Gilhar et al., 2011). Recently, it was reported that PIP_2_ applied through patch pipettes in whole-cell recordings significantly reduced Kv1.3 currents in Jurkat T cells and this regulation may be significant for the maintenance of T lymphocyte activation in immune responses (Matsushita et al., 2009). However, the mechanisms of this modulation need to be further explored.

Contrary to previous reports on Kv channel modulation by PIP_2_, Kruse et al. (2012) recently tested a long list of Kv channels and found most of them insensitive to PIP_2_; this list includes Kv1.1/Kvβ1.1, Kv1.3, Kv1.4, Kv1.5/Kvβ1.3, Kv2.1, Kv3.4, Kv4.2, and Kv4.3 (with different KChIPs). To test the effect of PIP_2_ on Kv channels this group used three different strategies to deplete PIP_2_ in intact cells, activation of the G protein-coupled muscarinic receptor M1, a zebrafish voltage-sensitive lipid 5-phosphatase (Dr-VSP), or an engineered fusion protein carrying both lipid 4-phosphatase and 5-phosphatase activity (pseudojanin). Kruse and colleagues offer some explanations for the discrepancy between previous experiments and their results, principally relying on the different strategies used to modulate the PIP_2_ concentrations in the membrane. While previous groups applied exogenous PIP_2_ to inside-out excised patches risking to increase PIP_2_ concentration to supramaximal levels, this group transiently depleted PIP_2_ in the membrane of intact cells maintaining all the constituents of the cell near normal conditions (Hilgemann, 2012; Kruse et al., 2012).

**Figure 1:**
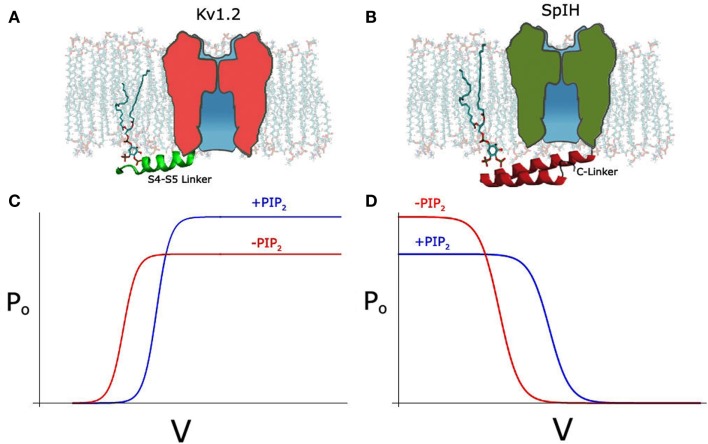
**(A)** Schematic depicting PIP_2_ interacting with S4–S5 linker (green) in the Kv1.2 channel, with linker shown in cartoon representation. **(B)** Schematic depicting PIP_2_ interacting with A′ helix of C-linker (red) in the SpIH channel, adapted from Flynn and Zagotta (2011). **(C)** Hypothetical Po-V curve of Kv1.2 showing depolarizing shift and increase in Po after PIP_2_ (blue line). **(D)** Hypothetical Po-V curve of SpIH showing depolarizing shift and decrease in Po after PIP_2_.

Recently, we also examined the effects of PIP_2_ depletion on the voltage-dependent Kv1.2 channel using different approaches not only in inside-out patches but also in intact cells, using the voltage-sensitive phosphatase Ci-VSP. We obtained similar results to the Cav channels, namely a bidirectional regulation, where PIP_2_ depletion left-shifted the voltage dependence of activation, increasing current at the same time it decreased the open probability causing an overall decrease in the current level (Figure 1C; Rodriguez-Menchaca et al., 2012). These two effects were kinetically distinct and exhibited distinct molecular determinants and sensitivities to PIP_2_. The effect on the voltage dependence of activation proceeded through interactions of the S4–S5 linker that links the voltage sensor to the channel pore with PIP_2_ (Figure 1A). Gating current measurements revealed that PIP_2_ constrains the movement of the sensor via specific interactions of basic residues with PIP_2_ in the closed state. In summary, using a similar strategy as Kruse et al. (2012) to transiently deplete PIP_2_ in intact cells namely a voltage-sensitive phosphatase, we obtained contrasting results showing a dual effect on Kv1.2 and Shaker channels after PIP_2_ depletion. In contrast, Kruse et al. did not observe any effect on the current amplitude of several Kv channels after PIP_2_ depletion and did not test for changes in voltage dependency.

Consistent with our results, Abderemane-Ali et al. (2012) also reported a dual effect of PIP_2_ on Shaker potassium channels; PIP_2_ exerts a gain-of-function effect on the maximal current amplitude and a positive shift in the voltage dependence of activation, through an effect on the voltage sensor movement.

## Hyperpolarization-Activated HCN Channels

Hyperpolarization-activated Cyclic nucleotide-gated (HCN) channels unlike most voltage-gated channels open only in response to membrane hyperpolarization (Gauss et al., 1998; Ludwig et al., 1998; Santoro et al., 1998). The S1–S4 domain constitutes the voltage sensor again but with HCN channels the S4 segment moves inward upon membrane hyperpolarization (Mannikko et al., 2002; Bell et al., 2004). The coupling mechanism between this inward movement of the S4 and the opening of the gate, which is presumably located near the intracellular end of S6, is not clear. The cytoplasmic domain of HCN channels possesses a canonical cyclic nucleotide binding domain (CNBD). The CNBD is connected to the S6 segment through a 90-aa sequence called the C-linker (CL). Direct binding of cAMP or cGMP (cNMP) to the CNBD facilitates channel opening. At the macroscopic current level, the cNMP-dependent gating right shifts the voltage dependence of activation and increases the current amplitude (Robinson and Siegelbaum, 2003; Craven and Zagotta, 2006; Biel et al., 2009). The CL plays a dominant role in the coupling of cNMP binding to the channel opening.

Hyperpolarization-activated cyclic nucleotide-gated channel activity is also under the control of PIP_2_. PIP_2_ right shifts the hyperpolarization-dependent HCN channel activation, making the channel easier to open (Pian et al., 2006; Zolles et al., 2006; Flynn and Zagotta, 2011; Ying et al., 2011). This effect is remarkable – about 20 mV for mammalian HCN channels (HCN1, 2, and 4) and independent from the regulation by cAMP and cGMP. Direct evidence supporting these conclusions came from the experiments on inside-out membrane patches. Applying either native PIP_2_ or the soluble diC_8_-PIP_2_ produced a depolarizing shift in HCN channel activation (Pian et al., 2006; Zolles et al., 2006; Flynn and Zagotta, 2011). The right shift in the I–V relationship could be reproduced separately under conditions of saturating concentrations of cAMP, in mutant channels that do not bind to cNMP, or in the HCN ΔC channels, in which the CL-CNBD has been deleted. Therefore, the interaction between PIP_2_ and the transmembrane domains with their connecting loops of HCN channels are likely to be responsible for the voltage-dependent effect rather than the CL and CNBD, which are essential for cAMP-dependent gating. It has been suggested that most likely, PIP_2_ exerts its effect on voltage dependence by stabilizing the activated state of the voltage sensor, which could be through either a generalized effect on the local electrostatic environment by PIP_2_ or a specific interaction between the negatively charged head-group of PIP_2_ with positively charged residues in the S4 or its surroundings (Flynn and Zagotta, 2011).

SpIH, a HCN channel cloned from Sea Urchin, can be fully activated by cAMP but only partially by cGMP (Flynn et al., 2007). Unlike the mammalian HCN channels, SpIH shows a bidirectional regulation by PIP_2_ (Flynn and Zagotta, 2011). Like mammalian HCN channels it shows a right shift of the voltage dependence of activation (∼10 mV) in the presence or PIP_2_, albeit half the magnitude of that in the mammalian channels (Figure 1D). Unlike the mammalian HCN channels, SpIH inactivates quickly in response to a hyperpolarizing voltage step (Gauss et al., 1998). Binding of cAMP or cGMP relieves this voltage-dependent inactivation and markedly increases the macroscopic current amplitude. Independent from the positive effect on the voltage-dependent activation, PIP_2_ seems to have an inhibitory effect on the cGMP-induced current (Figure 1D). This inhibitory effect is clearly related with the efficacy of the agonists. The maximal current under saturating concentration of cGMP is only about half of that of cAMP. Consistently, PIP_2_ strongly inhibits the cGMP-dependent current but has a minimal effect on the cAMP-induced current.

Noticeably, as reported more than a decade ago, PIP_2_ has a strong inhibitory effect on CNG channels, which is homologous to HCN channel and the cNMP binding is obligatory for its opening (Womack et al., 2000; Kaupp and Seifert, 2002). Several studies have produced atomic resolution structures of the HCN cytoplasmic C-terminal domains including that from SpIH channel (Zagotta et al., 2003; Flynn et al., 2007; Xu et al., 2010). Mutagenesis studies of the SpIH channel have identified several positively charged residues in the CL that contribute to the inhibitory effect by PIP_2_ (Figure 1B). It has been suggested that through these specific electrostatic interactions, PIP_2_ inhibits the channel opening by stabilizing the conformation of the CL that underlies the closed channel state in the absence of agonists (Flynn and Zagotta, 2011).

## Concluding Remarks

The bidirectional effects of PIP_2_ in three very different voltage-sensitive channels is quite remarkable. In the depolarization-activated Cav2.1 (Wu et al., 2002) and Kv1.2 (Rodriguez-Menchaca et al., 2012) studies, PIP_2_ right-shifted the voltage dependence of activation (inhibitory) while it prevented rundown by stabilizing the open probability of the channel (stimulatory). In the hyperpolarization-activated HCN channel from the sea urchin (SpIH) PIP_2_ right-shifted the voltage dependence of activation (stimulatory), while it inhibited the cGMP-induced activation (inhibitory). In Kv1.2, PIP_2_ stabilized the inactive state of the voltage sensor by binding basic residues in the S4–S5 linker and the N terminus that could only coordinate PIP_2_ in the closed state of the channel. In the SpIH HCN channel it was CL basic residues that accounted for the non-voltage-dependent effects of PIP_2_. In both cases these molecular determinants affected only one or the other of the dual effects of PIP_2_. These results from these very different channels (Cav, Kv, and HCN) suggest that the mechanism by which PIP_2_ regulates channels sensitive to voltage may be conserved.

What might be the physiological significance of the dual regulation of certain voltage-gated ion channels that we have highlighted in this review? We speculate that in channels that are dependent on voltage but also to other modulatory intracellular signals, a balance needs to be achieved to regulate gating in a coordinated manner. PIP_2_ and the S4–S5 linker are both perfectly positioned at the interface of the membrane to the cytosol to integrate cytosolic signals (e.g., cyclic nucleotides, Ca^2+^, etc.) with the movement of the transmembrane voltage sensor.

## Conflict of Interest Statement

The authors declare that the research was conducted in the absence of any commercial or financial relationships that could be construed as a potential conflict of interest.
